# Mental health service use among pregnant and early postpartum women

**DOI:** 10.1007/s00127-022-02331-w

**Published:** 2022-07-29

**Authors:** Leonie Lee-Carbon, Selina Nath, Kylee Trevillion, Sarah Byford, Louise M. Howard, Fiona L. Challacombe, Margaret Heslin

**Affiliations:** 1 Section of Women’s Mental Health, Institute of Psychiatry, Psychology & Neuroscience, King’s College London, London, UK; 2 Population, Policy and Practice Research & Teaching Department, University College London, London, UK; 3 King’s Health Economics, Institute of Psychiatry, Psychology & Neuroscience, King’s College London, London, UK

**Keywords:** Perinatal mental health, Mental health service use, Pregnancy, Postpartum

## Abstract

**Purpose:**

To explore the proportion and characteristics of women with a mental disorder who have contact with mental health services during pregnancy and the postnatal period in a maternity service in London.

**Methods:**

Data from the WEll-being in pregNancy stuDY (WENDY), a prospective cohort study, were used. Women were recruited at their first appointment for antenatal care and assessed for mental disorders using the Structured Clinical Interview DSM-IV Axis I/II Disorders for Research. Clinical, sociodemographic and psychosocial characteristics were collected. Mental health service use data were collected for the period from study entry to 3 months postpartum.

**Results:**

Two hundred women met diagnostic criteria for a mental disorder. Fifty-five (34%) of these had at least one contact with mental health services. Moderate depression (OR 7.44, CI 2.03–27.28, *p* < 0.01), severe depression (OR 10.5, CI 2.68–41.12, *p* < 0.01), past psychiatric hospital admission (OR 3.76, CI 1.05–13.44, *p* < 0.05), symptoms of anxiety (OR 3.95, CI 1.86–8.37, *p* < 0.001) and perceived low levels of social support (OR 0.43, CI 0.18–1.01, *p* = 0.05) were associated with an increased likelihood of contact with mental health services in univariate analyses. However, only moderate (OR 5.92, CI 1.31–26.78, *p* = 0.02) and severe depression (OR 6.04, CI 1.08–33.72, *p* = 0.04) remained significant in the multivariate regressions analyses.

**Conclusion:**

Only a third of women with a diagnosable mental disorder at their first antenatal appointment had any contact with mental health services during pregnancy or up to 3 months postpartum. Further research is warranted to elicit perinatal women’s views about the potential barriers to accessing professional mental health care.

## Introduction

Estimates suggest that up to one in five women experience a mental disorder during the perinatal period, that is, during pregnancy and the first 12 months following childbirth [1]. However, globally, there is a disparity between the underlying prevalence of perinatal mental disorder and the proportion of women who access mental health services; significant numbers of perinatal women are not receiving the mental health treatment they need [2]. Indeed, one study reports that only 35% of women who met diagnostic criteria for depression accessed professional treatment during the perinatal period [3].

The burden of untreated perinatal mental disorders can be observed at several levels. Psychiatric illness is one of the leading causes of maternal morbidity and mortality, with several links made between mental disorders during pregnancy and obstetric complications, impaired parent–infant bonding, delayed child development and paternal psychological distress [4–7]. Moreover, on a societal level, estimates suggest that poorly managed and untreated perinatal mental disorders in the UK [8] costs £8.1 billion for each 1-year cohort of birth [9]. Given the ramifications of unmet need, national guidelines in the UK (National Institute of Care and Excellence- NICE) recommend that mental health services promote equal access to care by offering timely and evidence-based mental health screening and treatment during the perinatal period [NICE, 10].

Standardised screening tools, such as the Whooley questions, are recommended for screening symptoms of depression and the Generalised Anxiety 2 Scale was previously recommended to screen for anxiety [11]. A positive screen on either instrument indicates an elevated probability of caseness, i.e. of mental disorder and warrants further assessment [12], with referrals being made to appropriate services, in accordance with the woman's needs. Psychotropic medication, talking therapies and care coordination are some of the interventions clinicians can employ to support perinatal women manage specific mental disorders [11].

To further promote equity in access, regional coverage of specialist (secondary care) perinatal mental health services within the UK has expanded; in 2018, 24% of areas in the UK still lacked services compared to the 50% in 2015 [13, 14]. The unprecedented expansion in services warrants an evaluation of the patterns of mental health services contact during the perinatal period. To the best of the author’s knowledge, there are no quantitative-based published findings, conducted in the UK, which has explored this research area (Lee-Carbon, Doctoral Thesis). Research conducted internationally indicates that perinatal women who, before pregnancy, had a mental disorder or contact with mental health services were more likely to have contact again during the perinatal period [3, 15–23]. Such patterns may occur because women with these prior experiences are more astute to recognising symptoms of mental disorder and, particularly among those with a serious mental disorder (e.g. psychosis), may continue care with the same mental health-care provider during pregnancy [16, 22].

The international body of research suggests that women experiencing financial stress (e.g. material deprivation and the scarcity of money to pay for treatment or transportation) or from an ethnic or racial minority backgrounds are less likely to have contact with mental health services compared to those without this sociodemographic profile [15–17, 23–33]. The practical concerns regarding the affordability of health care, language barriers to communicate with mental health services, perceptions of mental health services and the role of stigma are some of the explanations to describe this pattern of contact with mental health services [17, 26, 34].

Some findings may not be generalisable to the UK given the variations in health-care provision and public health spending investments. The international body of literature also has some methodological limitations: for example, measuring the intention to seek professional help as opposed to measuring actual contact with mental health services. Moreover, research has heavily relied upon screening tools to identify perinatal mental disorders, which lack the accuracy and specificity of a structured interview (i.e. the Structured Clinical Interview for DSM Disorders) or clinician diagnoses using international criteria (DSM or ICD) [12]. Collectively, such approaches make it challenging to identify the level of unmet need among a cohort of perinatal women.

Information on who does and does not contact mental health services in the perinatal period within the UK context could inform health-care commissioners of the unmet mental health needs as services expand and provide guidance to non-mental health professionals (e.g. midwives, health victors, obstetric gynaecologists) involved in the woman’s care throughout the perinatal period about women most at risk of not contacting mental health services. However, the limitations of previous research are likely to bias the findings. A study addressing this issue, but improving on the methodology by utilising a clinician-administered diagnostic assessment is needed.

This study sought to explore contact with mental health services during pregnancy and up to 3 months postpartum among a cohort of women who met diagnostic criteria for a mental disorder in their first trimester of pregnancy.

Specifically, we aimed to:

- (1) identify the proportion of women who had contact with any type of mental health service,
- (2) describe the type of mental health services women had contact with,
- (3) explore the clinical, sociodemographic and psychosocial factors associated with contact with mental health services during the study period.

## Methods

### Study design and ethical approval

Secondary analyses were conducted on an existing dataset: a cohort study called the WEll-being in pregNancy stuDY (WENDY) [12]. The WENDY Study was granted ethical and governance approval from the Research Ethics Committee (reference: 14.LO.0075) on 14/02/2014. Ethical approval was sought from King’s College London to address the specific research objectives of the current study; the Psychiatry, Nursing and Midwifery Research Ethics Panel approved the application on 28/02/2019 (reference: LRS- 18/19–8451).

### Participants and procedures

Recruitment for the WENDY Study was based in South London and took place between 10 November 2014 and 30 June 2016. Women were asked the Whooley questions by their midwife. Women who had a negative Whooley status (unlikely to have major depression) were randomly invited to participate 1:4, then 1:6, compared to those with a positive Whooley status (an indicator of major depressive symptoms) who were all invited. Women were approached by a researcher to discuss participation in the study, either at their antenatal booking appointment or shortly thereafter via telephone (approximately, 10–12 weeks gestation) [12]. Where applicable, interpreters translated the study aims and gained informed consent, to support women who may otherwise encounter language barriers to participate in the study. Written informed consent was gathered at the baseline research interview.

During the baseline research interview (mean gestation 14-weeks), conducted within 3 weeks of the woman’s antenatal booking appointment, participants underwent a diagnostic clinical interview and completed sociodemographic and standardised self-report measures which were translated if necessary [12]. Participants were followed up twice; 28 weeks gestation and 3 months postpartum) to explore contact with mental health services.

Women were excluded if they were under the age of 16, did not complete the Whooley questions or terminated their pregnancy before the baseline research interview. A detailed description of the methodology of the WENDY Study is published elsewhere [12].

Women were excluded from the current study if they were participating in the ‘Antenatal guided self-help for women’ (DAWN) randomised controlled trial (part of the same programme of work as the WENDY Study) exploring the efficacy of a guided self-help intervention to treat mild–moderate symptoms of depression [35]. This is because randomisation to the DAWN intervention involved treatment by mental health services.

### Measures

#### Measure of perinatal mental disorders

The Structured Clinical Interview DSM-IV Axis I/II Disorders for Research (SCID I/II R) is a researcher-administered semi-structured interview used to assess the presence of a mental health disorder [36]. The following modules were administered by researchers at the first research appointment: mood episodes, mood disorders, anxiety disorders, eating disorders (SCID I) and the subsection for borderline personality disorder (SCID II). Researchers undertook formal training to administer the interview, and in cases of ambiguity, a consensus diagnosis was achieved under the supervision of the study principal investigator, a psychiatrist [12]. For data analytical purposes, participants were grouped in one of the two following categories:

1. ‘SCID-positive women’—women who met criteria for any current mental disorder.
2. ‘SCID-negative women’—women without a current mental disorder.

For descriptive purposes, diagnoses were defined as: ‘Depressive Disorder’ (major depression); ‘Any Anxiety Disorder’ (panic, agoraphobia, social, specific, obsessive–compulsive, generalised, post-traumatic stress, and acute stress disorders, as per DSM-IV guidelines); ‘Any Eating Disorder’ (atypical anorexia, bulimia, binge eating, purging, and non-specified); ‘Bipolar Disorder’ (manic or hypomanic); ‘Mixed Anxiety and Depression’; ‘Borderline Personality Disorder’ and ‘Comorbid Condition’ (which includes a combination of 2 or more of the aforementioned categories).

### Service use measure

A modified version of the Adult Service Use Schedule (AD-SUS) was used to collect information on the use of health and social care services. The AD-SUS was collected to allow an economic evaluation to be conducted within the WENDY Study, not for exploring contact with mental health services during pregnancy as it is used here. It was developed for use in a range of adult mental health populations [8, 37, 38] and was modified for the purpose of this study to cover antenatal services as well as general health and social services, both for the mother and the infant. The AD-SUS was completed by research assistants in an interview with participants at 28 weeks gestation and 3 months postnatally. The 28 weeks gestation AD-SUS covered the period from the antenatal booking appointment (baseline) to the 28 -weeks gestation follow-up interview. The AD-SUS at 3 -months postnatal covered the period from the date of the 28 -weeks gestation interview to the 3 month postnatal interview.

Participants were asked to report any contacts with a range of health and social care services, including all mental health services. Specifically, questions in the AD-SUS that focused on mental health services were as follows:

1. Have you had any contact with any of the following community services:
  (i) psychological therapies/talking therapies low intensity: groups, workshops and guided self-help (written materials with regular support on the phone or face to face)?
  (ii) psychological therapies/talking therapies (IAPT) high intensity: counselling CBT or other therapy, weekly face to face individual sessions?
  (iii) community psychiatric nurse?
  (iv) clinical psychologist/counsellor?
  (v) community psychiatrist?
  (vi) perinatal psychiatric/home treatment team?
2. Have you had any outpatient appointments for your mental health?
3. Have you been admitted to hospital for your mental health?

Answers were dichotomised to ‘yes’ or ‘no’. An answer of yes to one or more of the above questions was classified as a ‘contact’ with mental health services. Participants were considered not to have contacted mental health services if they responded ‘no’ to all the above questions. Contact types were grouped into three categories: primary care (low- and high-intensity IAPT); secondary care (community psychiatric nurses, clinical psychologists or counsellors, community psychiatrists, perinatal psychiatrists or home treatment teams); and inpatient services (hospital stays for mental health).

### Independent variable measures

Clinical, sociodemographic and psychosocial information was collected at the baseline interview.

Three clinical measures were used to explore clinical symptoms including:

1. The Edinburgh Postnatal Depression Scale (EPDS) is a ten-item self-report questionnaire developed to measure the presence and severity of depressive symptoms during the perinatal period [39]. In this study, the symptom severity scale was used to categorise no or minimal (scores 0–6), mild (scores 7–13), moderate (scores 14–19) and severe (scores 20–30) symptoms [40]. Additionally, the threshold score of 13 or above (which identifies those with elevated depressive symptoms) was applied.
2. The Generalized Anxiety Disorder Scale (GAD-2) is a two-item self-report scale to screen for common anxiety disorders including post-traumatic stress disorder (PTSD), generalised, panic and social anxiety [41]. The GAD-2 has a sensitivity rating of 86% and specificity of 83% when screening for generalised anxiety disorder, and for any anxiety disorders ratings are 65% and 88%, respectively, using the threshold of three or above [41].

As part of the baseline interview, researchers collated the remaining clinical information: ‘history of psychiatric admission’ and ‘history of deliberate self-harm or attempted suicide’ with answers limited to ‘yes’ or ‘no’. Sociodemographic information including age, employment status, ethnicity, household income, the highest level of education, immigration status and living arrangements were also collected as categorical variables.

Psychosocial information was derived from the Social Provisions Scale (SPS). This 24-item self-report questionnaire was developed to examine the responder's experience of social provision [42]. Participants rate their current relationship experiences on a scale ranging between 1 “strongly disagree” to 4 “strongly agree”. The scores are totalled, and higher scores indicate greater perceived social support. The questionnaire has been used in perinatal research and has shown good reliability and construct validity [42, 43]. The current study generated quartiles from the total SPS score to characterise the level of perceived social support as reported in [44]. To do this, a total score was calculated and ascendingly ordered; participants with total scores at the lower end of the scale scores (25% or lower) formed the bottom quartile indicating low levels of perceived social support. Those scoring in the top three quartiles were considered to have higher levels of perceived social support.

### Statistical analysis

STATA version 15.1 was used to conduct analyses.

Women with missing data were also excluded from the main analysis. Key sociodemographic and baseline clinical characteristics for those with and without missing data were explored descriptively using frequencies and percentages to assess for any biases between the two groups. The proportion of women who reported contacting mental health services between the antenatal booking appointment and 28 weeks gestation and between 28 weeks gestation and 3 months postpartum, and overall, and the types of services that women reported using were explored with frequencies and percentages. Where women accessed more than one service, the service which provided care for the highest level of need was reported in the order inpatient, outpatient and primary care (i.e. if a patient contacted community and inpatient mental health services, then ‘inpatient service’ was recorded as the type of service accessed). Bivariate logistic regressions were used to test the relationship between mental health service use (yes/no) and baseline clinical, sociodemographic and psychosocial characteristics. Statistically significant variables (*p* value < 0.05) were included in a multiple regression analysis.

## Results

### Sample

A total of 545 participants were recruited to the WENDY Study. Of these, 42 were also recruited and randomised to the DAWN trial and were thus excluded from the current study. Of the remaining 503 participants, 494 undertook the full SCID IV assessment with 40% (*n* = 200) meeting SCID criteria for a mental disorder and hereafter referred to as ‘SCID-positive’ participants.

### Data availability

Of the 200 participants with an SCID-positive diagnosis, 58.5% (*n* = 117) had complete data on the measures required for the final multiple regression analyses. Table 1 describes the sociodemographic and clinical characteristics of those with complete and those with incomplete data. Notable differences included ethnicity, academic attainment, relationship status, income, GAD-2 symptoms, depressive disorder, insecure immigration and first language. Women who identified as being from a Black background, low education attainment (secondary education or lower), on the lowest income bracket (up to £5,475), not in a relationship, being in insecure immigration status, having English as a second language, GAD positive or diagnosed with a depressive disorder were more likely to have incomplete data. In comparison, women from a White background or earning £61,000 or more were more likely to have complete data. The reduced data availability from women from low-income and marginalised backgrounds, or those presenting with depression or increased anxiety may indicate some biases.

**Table 1:** Comparison between the SCID-positive women who had complete data on the measures required for the final multiple regression analyses and those who did not

Sociodemographic and clinical factors	Complete data^a^ *N* = 117 *n* (%)			Incomplete data *N* = 83 *n* (%)	
**Age group**					
16–24 years	19		(16.2)	20	(24.1)
25 years and over	98		(83.8)	63	(75.9)
**Ethnicity**					
White	62		(53.0)	29	(34.9)
Black	36		(30.8)	36	(43.4)
Asian	6		(5.1)	5	(6.0)
Mixed ethnicity	5		(4.3)	5	(6.0)
Other ethnic category	8		(6.8)	8	(9.6)
**Highest level of academic attainment**					
GCSE or Lower	14		(12.0)	21	(25.3)
A-Level or Equivalent	33		(28.2)	23	(27.7)
University Degree or above	70		(59.8)	39	(47.0)
**Employment status**					
Employed	75		(65.2)	51	(61.5)
Unemployed	40		(34.8)	32	(38.6)
**First language**					
English	78		(66.7)	47	(56.6)
Other	39		(33.3)	36	(43.4)
**Immigration status**					
Secure	99		(84.6)	63	(75.9)
Insecure	18		(15.4)	20	(24.1)
**Income**^b^					
£0–£5475	13		(13.8)	18	(31.0)
£5476–£14,999	9		(9.6)	5	(8.6)
£15,000–£30,999	17		(18.1)	14	(24.1)
£31,000–£45,999	9		(9.6)	5	(8.6)
£46,000–£60,999	14		(14.9)	8	(13.8)
£61,000 +	32		(34.0)	8	(13.8)
**Relationship status**					
Single, separated, divorced, widowed	16	(13.7)	20	(24.1)	
In a relationship	101	(86.3)	63	(75.9)	
**EPDS severity**					
No or min depression	21	(18.0)	6	(11.5)	
Mild depression	51	(43.6)	20	(38.5)	
Moderate depression	26	(22.2)	14	(26.9)	
Severe depression	19	(16.2)	12	(23.1)	
**GAD-2 severity**					
Negative ≤ 2	73	(62.4)	24	(50.0)	
Positive ≥ 3	44	(37.6)	24	(50.0)	
**History of deliberate self-harm or suicide attempt**					
No	86	(73.5)	65	(78.3)	
Yes	31	(26.5)	18	(21.7)	
**Structured clinical interview diagnosis**					
Depressive disorder	32	(27.4)	33	(39.8)	
Any anxiety disorder	30	(25.6)	18	(21.7)	
Any eating disorder	3	(2.6)	3	(3.6)	
Bipolar types	1	(0.9)	1	(1.2)	
Mixed anxiety depression	5	(4.3)	1	(1.2)	
Borderline personality disorder	–	–	1	(1.2)	
Comorbid	46	(39.3)	26	(31.3)	
**Parity**					
One child	62	(53.0)	41	(49.4)	
More than one child	55	(47.0)	42	(50.6)	
**History of psychiatric admission**					
No	108	(92.3)	80	(96.4)	
Yes	9	(7.7)	3	(3.6)	

^a^Complete data includes the following: contact with mental health services (AD-SUS), Edinburgh Postnatal Depression Scale (*EPDS*), Generalized Anxiety Disorder 2-item (*GAD-2*), history of psychiatric admission and Social Provision Scale

^b^Some household income data were missing (*n* = 94 complete data; *n* = 58 incomplete data)

### DSM-IV diagnoses

**Fig. 1:**
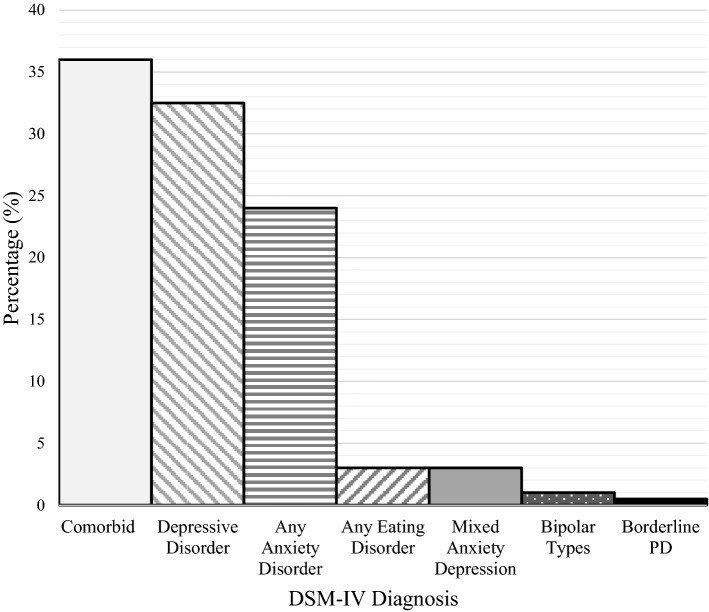
Frequency of DSM-IV diagnosis

DSM-IV diagnoses are presented in Table 1 and Fig. 1. Of the 200 included participants, 36% (*n* = 72) met the criteria for more than one DSM-IV mental disorder and were therefore categorised within the ‘comorbid’ group. Of those within the comorbid group, 17% (*n* = 12) met the criteria for borderline personality disorder with depression or anxiety and 14% (*n* = 10) of participants with an eating disorder and depression or anxiety. Women with a single DSM-IV diagnosis most commonly met the criteria for a depressive disorder (32.5%, *n* = 65).

### Contact with mental health services and type of services used

Types of mental health services accessed by participants over the 28 weeks gestation and 3 months postpartum time periods are reported in Table 2. Thirty-four percent (*n* = 55) of women with an SCID diagnosis at baseline had contact with mental health services over the whole follow-up period, 23% (*n* = 40) between baseline and 28 weeks gestation and 24% (*n* = 40) between 28 weeks gestation and 3 months postpartum. Of those who had contact with mental health services over the course of the study period, 7.3% (*n* = 4) had contact with inpatient mental health services as their highest level of care, 69.1% (*n* = 38) had contact with secondary mental health services as their highest level of care and 23.6% (*n* = 13) had contact with primary care mental health services only. Very few participants (6.3%, *n* = 3) accessed more than one type of mental health service; those who did, all accessed secondary and inpatient mental health services.

**Table 2:** Type of mental health services accessed across the study period

Type of mental health services contact	Time period					
	Baseline to 28 weeks gestation (T1) *N* = 40		28 weeks gestation to 3 months postpartum (T2) *N* = 40		Overall (T1 + T2 combined) *N* = 55	
	*n*	%	*n*	%	*n*	%
Primary care						
Low-intensity therapy	2	(5.0)	2	(5.0)	2	(3.6)
High-intensity therapy	10	(25.0)	8	(20.0)	11	(20.0)
Secondary care						
Community psychiatric nurse	0	0	1	(2.5)	1	(1.8)
Clinical psychologist or counsellor	9	(22.5)	3	(7.5)	9	(16.4)
Community psychiatrist	0	0	1	(2.5)	1	(1.8)
Perinatal psychiatric or home treatment team	18	(45.0)	22	(55.0)	27	(49.1)
Other mental health outpatient	0	(0)	1	(2.5)	1	(1.8)
Inpatient care						
Inpatient admission	1	(2.5)	3	(7.5)	4	(7.3)

### Predictors of mental health service use among SCID positive participants

#### Univariate regression analyses

Unadjusted logistic regression analyses for key baseline clinical, sociodemographic and psychosocial characteristics revealed that perceived social support, EPDS category (mild, moderate, severe), past psychiatric admission and GAD-2 status were all significantly associated with mental health service contact (Table 3).

**Table 3:** Univariate regression analyses of contact with mental health services and sociodemographic, psychosocial or clinical characteristics

Sociodemographic, psychosocial, and clinical characteristics	No contact with mental health services		Contact with mental health services		OR (unadjusted)	95% CI	*p* value	Likelihood Ratio overall *p* value
	*n*	%	*n*	%				
Age group (*n* = 162)								
16–24 years	19	(17.8)	9	(16.0)	(reference)			0.05 (1), *p* = .82
25 years and over	88	(82.2)	46	(84.0)	1.10	0.46–2.63	0.82	
Employment status^a^ (*n* = 160)								
Employed	76	(71.7)	31	(57.4)	(reference)			**3.24 (1), *p* = 0.07**
Unemployed & other	30	(28.3)	23	(42.6)	1.88	0.94–3.70	0.07	
Ethnic minority (*n* = 162)								
White	48	(44.9)	29	(52.7)	(reference)			1.42, (4), *p* = 0.84
Black	40	(37.4)	16	(29.1)	0.66	0.32–1.39	0.32	
Asian	6	(5.6)	3	(5.5)	0.83	0.19–3.57	0.20	
Mixed ethnicity	5	(4.7)	2	(3.6)	0.66	0.12–3.64	0.12	
Other ethnicity	8	(7.5)	5	(9.1)	1.03	0.31–3.46	0.31	
Highest level of academic attainment (*n* = 162)								
GCSE or lower	15	(14.0)	8	(14.6)	(reference)			0.33, (2), *p* = 0.85
A-level or equivalent	27	(25.2)	16	(29.1)	0.85	0.39–3.20	0.85	
University degree or above	65	(60.8)	31	(56.4)	0.82	0.34–2.33	0.82	
Income level^a^ (*n* = 127)								
£0–£5475	13	(15.9)	9	(20.0)	(reference)			5.60, (5) *p* = 0.35
£5476–£14,999	7	(8.5)	4	(8.9)	0.83	0.19–3.68	0.8	
£15,000–£30,999	11	(13.4)	11	(24.4)	1.44	0.44–4.76	0.55	
£31,000–£45,999	7	(8.5)	6	(13.3)	1.24	0.31–4.93	0.76	
£46,000–£60,999	14	(17.1)	5	(11.1)	0.52	0.14–1.95	0.33	
£61,000 +	30	(36.6)	10	(22.2)	0.48	0.16–1.46	0.2	
Immigration status **(***n* = 162**)**								
Secure	87	(81.3)	48	(87.0)	(reference)			0.97, (1), *p* = 0.33
Insecure	20	(18.7)	7	(12.7)	0.63	0.25–1.61	0.34	
Relationship status (*n* = 162)								
Single, separated, divorced, widowed	16	(15.0)	11	(20.0)	(reference)			0.65, (1), *p* = 0.42
In a relationship	91	(85.1)	44	(80.0)	0.70	0.30–1.64	0.42	
Social provision (SPS)^a^ (*n* = 121)								
Bottom percentile	14	(18.2)	15	(34.1)	(reference)			3.79, (1), ***p* = 0.05**
Top three percentile	63	(81.8)	29	(65.9)	0.43	0.18–1.01	**0.05**	
Edinburgh Postnatal Depression Scale (EPDS) category * (*n* = 134)								
No or minimal depressive symptoms	21	(24.4)	4	(8.3)	(reference)			**27.48, (3),** ***p* < 0.001**
Mild depressive symptoms	45	(52.3)	11	(22.9)	1.28	0.37–4.51	0.7	
Moderate depressive symptoms	12	(14.0)	17	(35.4)	7.44	2.03–27.28	**< 0.001**	
Severe depressive symptoms	8	(9.3)	16	(33.3)	10.50	2.68–41.12	** < 0.001**	
History of deliberate self-harm or suicide attempt (*n* = 138)								
No	81	(75.7)	40	(72.7)	(reference)			0.17, (1), *p* = 0.68
Yes	26	(24.3)	15	(27.3)	1.17	0.56–2.45	0.68	
Past psychiatric admission (*n* = 162)								
No	103	(96.3)	48	(87.3)	(reference)			**4.34, (1),** ***p* < 0.05**
Yes	4	(3.7)	7	(12.7)	3.76	1.05–13.44	**0.04**	
Generalized Anxiety Disorder 2-item (GAD-2)^a^ (*n* = 132)								
GAD negative	62	(73.8)	20	(41.7)	(reference)			**13.35, (1), *p* < 0.001**
GAD positive	22	(26.2)	28	(58.3)	3.95	1.86–8.37	** < 0.001**	
More than 1 child (*n* = 162)								
No	61	(57.0)	23	(41.8)	(reference)			**3.37, (1), *p* = 0.07**
Yes	46	(43.0)	32	(58.2)	1.84	0.96–3.56	**0.07**	

^a^There were additional missing values for these variables, as not all women completed these measures

The odds of women with moderate or severe symptoms of depression accessing mental health services were 7.44 (CI: 2.03–27.28, *p* < 0.001) and 10.5 (CI: 2.68–41.12, *p* < 0.001), respectively, as measured by the EPDS, compared to women with no or minimal symptoms. Women who scored positively on the GAD-2 questionnaire (i.e. presenting with symptoms of anxiety) were at increased odds (OR: 3.95, CI: 1.86–8.37, *p* < 0.001) of contacting mental health services compared to those who were GAD-2 negative, indicating a positive association between contact with mental health services amongst women who self-reported higher levels of anxiety symptoms. Women who had previously experienced a psychiatric admission were at higher odds (OR 3.76, CI: 1.05–13.44, *p* < . 05) of contacting mental health services during the study period when compared to those without a psychiatric admission in their lifetime. Also, women who scored in the top three quartiles on the social provision scale, and therefore had higher levels of perceived social support, were at lower odds (OR 0.43, CI: 0.18–1.01, *p* = 0.05) of contacting mental health services when compared to women in the bottom percentile. This indicates that women who perceived greater levels of social support were significantly less likely to contact mental health services during the study period when compared to women who perceived comparatively low levels of social support.

There were some between-group differences in employment status and contact with mental health services. Indeed, women not in employment were more likely (OR 1.88, CI: 0.94–3.70, *p* = 0.07) to have contact with mental health services, when compared to employed women; though this difference was not statistically significant. There were also between-group differences in the number of children women had and contact with mental health services. Women with more than one child were slightly more likely (OR 1.84, 0.96–3.56, *p* = 0.07) to have contact with mental health services when compared to women with one child, though the differences were non-significant.

#### Multiple regression analyses

Multiple regression analyses were conducted to explore the relationship between key independent variables that were significant in the univariate model (EPDS category, history of psychiatric admission, GAD-2 and social provision), and the outcome variable in one regression model. Table 4 indicates that moderate (OR = 5.92, CI: 1.31–26.78, *p* = 0.02) and severe depression (OR 6.04, CI: 1.08–33.72, *p* = 0.04), as measured by the EPDS, remained the only significant predictors of mental health services use.

**Table 4:** Multiple regression analysis of contact with mental health services and statistically significant sociodemographic, psychosocial and clinical characteristics

Variables statistically significant in the univariate analysis	OR	95% CI	*p* value
Edinburgh Postnatal Depression Scale (EPDS) category			
No or minimal symptoms	(reference)		
Mild depression	1.41	0.34–5.80	0.64
Moderate depression	5.92	1.31–26.78	0.02^*^
Severe depression	6.04	1.08–33.72	0.04^*^
History of psychiatric admission			
No	(reference)		
Yes	3.17	0.68–14.89	0.14
Social provision			
Bottom quartile	(reference)		
Top three quartiles	0.75	0.27–2.10	0.58
Generalized Anxiety Disorder 2-item (GAD-2)			
GAD-2 negative	(reference)		
GAD-2 positive	1.56	0.60–4.07	0.36

^*^p < 0.05

## Discussion

This study found that only 34% of women who had a diagnosable mental disorder in the first trimester of pregnancy had contact with mental health services from antenatal booking to 3 months postpartum. These findings are comparable to those reported in another study in the USA who report, among their cohort of perinatal women with or without any type of diagnosed perinatal mental disorder, 38% visited mental health services during the study period [32].

This study also found of those who did have contact, that the majority of women had contact with secondary mental health services, which may reflect the investment and expansion in specialist secondary care perinatal mental health services in the UK over recent years [14]. It was beyond the scope of the study to assess whether participants received the appropriate level of care according to their needs or whether the care was effective.

In relation to factors associated with the use of mental health services, this study found that moderate or severe self-reported symptoms of depression as reported by the EPDS were predictive of contact with mental health services and this variable maintained significance in the multiple regression. These findings are corroborated by other research that indicates increased severity in perinatal depressive symptoms is associated with help-seeking behaviours [45–47]. One study conducted in Israel reported that the odds of using professional psychological services increased by 45% of every additional point on the EPDS [47]. Increased symptom severity may also suggest a greater impact on daily functioning, thus potentially exposing the need for professional help to both health-care professionals and patients alike.

There is also evidence from this study that self-reported and elevated anxiety symptoms, low social support, or a history of psychiatric admission have a direct and positive relationship with contact with mental health services, although only in univariate analyses.

Our findings indicate that women who perceived high levels of social support were less likely to have contact with mental health services. Those who perceive high levels of support from their social networks may feel that their emotional needs are being met and may not desire support from professional services. However, some findings indicate that women who do not perceive encouragement to access mental health treatment from their partner or family, are more likely to identify barriers and have low intention to engage in treatment [48, 49]. Further research is warranted on the potential role of stigma and perceptions of mental health services during the perinatal period amongst women and family members more broadly.

A positive association between past mental disorders and perinatal mental health services use has been documented in studies conducted outside of the UK [15–17]. Past psychiatric admission may speak to the presence and severity of mental disorder before pregnancy; those who had a significant mental disorder to warrant hospitalisation before pregnancy could be more astute in recognising relapse in mental state and thus may be more likely to seek help or be offered it [17]. Within the context of expanding perinatal mental health services, further research could explore whether pregnant women previously admitted to a psychiatric ward are prophylactically referred to secondary care mental health services for monitoring.

This study did not identify any differences in the demographic characteristics between those who did and did not have contact with mental health services. Demographic factors such as young maternal age, ethnic or racial minority status and low income have frequently been reported as barriers to perinatal access to mental health services in similarly designed studies conducted outside of the UK [15, 32, 46]. One explanation for the difference is that our study was conducted in a diverse inner-city setting and, in accordance with their participation in the study and the prospective study design, all women underwent a diagnostic assessment and were signposted to services if warranted. Qualitative findings in the UK suggest that the level or confidence in basic mental health training and maternal demographics (e.g. level of deprivation or education, and ethnicity) may influence maternity staff’s decision to administer mental health screening assessments during routine antenatal appointments [50, 51]. If women with demographics typically underrepresented in mental health services are not screened during their standard perinatal appointments, it may delay or act as a barrier to accessing mental health services. A study in the USA suggests that when perinatal women are universally screened and offered an accessible mental health programme tackling barriers which women from an ethnic minority or low-income encounter, demographic disparities in service uptake dissipate [31].

### Strengths and limitations

The current study has several strengths, including being one of few published findings that explore patterns of access to mental health services based on a moderately large sample size within the UK, with a diverse sample that was representative of the local population [12]. These approaches enhance the generalisability of the findings to the local region particularly as a robust diagnostic assessment was used to identify a current mental disorder. Where needed, language interpreters were used to enable non-English speaking women to participate, and the prospective nature of the study design enabled us to make inferences regarding the direction of associations.

However, limitations must also be considered. Mental health diagnosis was measured once in the first trimester and not assessed at later points. Thus, some women could have recovered sufficiently to not need contact with mental health services meaning our estimate of the number of women who need contact with mental health services could be overestimated. Conversely, some women could have developed mental health difficulties after the first trimester of pregnancy or in the postpartum period and this is not detected in the study. Further, in the measurement of perinatal mental disorders, only the mood, anxiety and eating disorders section of the SCID I were used and the borderline personality disorder of the SCID II were used. This means that women with psychosis spectrum disorders, other personality disorders and other disorders will have been misdiagnosed as having no mental health condition. Moreover, since the primary purpose of the original study was to identify women with depression (this is a secondary data analysis of another study), the sampling strategy focussed on interviewing all women who were screened as positive for depression on the Whooley questions, and 1:4–1:6 of women who screened negative on the Whooley questions, it is likely that depression would be the most highly detected diagnosis and may be over represented in this study.

Also to consider is that all women who underwent a diagnostic assessment were signposted to services if warranted. This could have artificially increased the number of people in contact with mental health services, making our findings an overestimate. As the recruitment period of 2014–2016 in this study overlaps with the expansion of perinatal mental health services within the UK between 2015 and 2018, the estimates of service use from this study may no longer be accurate. Further, we measured contact with mental health services rather than treatment, or appropriate treatment, meaning that our estimate of the number of women who need mental health services input and received it could be underestimated. Additionally, we were not able to account for women who may have had their mental health treated by their GP since data was not collected on the reason for contacts with GPs, making it impossible to assess the proportion that were for mental health reasons compared to other reasons. In relation to this, the AD-SUS was not created specifically with this paper in mind, hence we had to make assumptions about the classification of certain mental health contacts. For example, counsellors were included as secondary care, but it is possible they were seen in primary care settings. Clinical psychologists can be primary care or secondary, e.g. they may work in IAPT but we chose to include them here in secondary care as we believed clinical psychologists seen in IAPT would be more likely to be reported as “IAPT” (high-intensity) services by the participants. In terms of analysis, only 58.5% of women with a SCID positive diagnosis had full data allowing them to be included in the multiple regression, and there were some differences between the group with and without complete data. This could have biased the results of the regression analyses. Additionally, no corrections were made for multiple testing.

## Conclusion

Just over one-third of women who met diagnostic criteria for a mental disorder in the first trimester of pregnancy sought professional help in the subsequent year. The findings emphasise the importance of universal mental health screening throughout the perinatal period to help detect those with or at most risk of developing a mental health disorder, and clear referral pathways to promote service use when needed. Findings from this study suggest that among women with a diagnosed perinatal mental disorder, those who indicate moderate to severe depressive symptoms on the EPDS are at increased odds of having contact with mental health services during pregnancy and up to 3 months postpartum. Considering this relationship, further psychoeducation might be indicated for health-care professionals and families alike, to raise awareness of the gravity and variation of perinatal mental disorder particularly when women do not self-report high depressive symptoms. Further research is warranted to understand the mental health outcomes and experiences of women who have contact with mental health services during the perinatal period to bring further context to this discussion.
